# Introducing *Women's Health Reports*

**DOI:** 10.1089/whr.2019.28999.edi

**Published:** 2020-01-20

**Authors:** Susan Kornstein, Robert Downs

**Affiliations:** Virginia Commonwealth University School of Medicine, Richmond, Virginia.

We are pleased to announce the launch of a new online open access journal, *Women's Health Reports. Women's Health Reports* is a companion journal to *Journal of Women's Health* and allows for an expansion of the literature into new territories that our flagship journal cannot accommodate due to space constraints imposed by the print format model. *Women's Health Reports* will provide a home for clinical papers, translational research, and health promotion and wellness articles covering a broad range of topics. Clinical case reports, basic research papers, and other articles that are regional in scope or of interest to specialized groups of readers will be a better fit for *Women's Health Reports*.

Readers will appreciate this new publication being open access, meaning that no subscription is required to access and read the content. For authors, publishing under a creative commons license allows for them to maintain ownership of their copyright. This model allows for a win-win on both sides and fosters a spirit of openness and collaboration. This spirit is important for driving research in our field forward as we aim to support advances in healthcare for women around the world.

